# The Plastisphere – Uncovering tightly attached plastic “*specific*” microorganisms

**DOI:** 10.1371/journal.pone.0215859

**Published:** 2019-04-23

**Authors:** Inga Vanessa Kirstein, Antje Wichels, Elisabeth Gullans, Georg Krohne, Gunnar Gerdts

**Affiliations:** 1 Alfred-Wegener-Institute Helmholtz Centre for Polar and Marine Research, Biologische Anstalt Helgoland, Helgoland, Germany; 2 University of Würzburg, Biocenter, Imaging Core Facility, Würzburg, Germany; University of Porto, PORTUGAL

## Abstract

In order to understand the degradation potential of plastics in the marine environment, microorganisms that preferentially colonize and interact with plastic surfaces, as opposed to generalists potentially colonising everything, need to be identified. Accordingly, it was hypothesized that i.) plastic “*specific*” microorganisms are closely attached to the polymeric surface and ii.) that specificity of plastics biofilms are rather related to members of the rare biosphere. To answer these hypotheses, a three phased experiment to stepwise uncover closely attached microbes was conducted. In Phase 1, nine chemically distinct plastic films and glass were incubated *in situ* for 21 months in a seawater flow through system. In Phase 2, a high-pressure water jet treatment technique was used to remove the upper biofilm layers to further, in Phase 3, enrich a plastic “*specific*” community. To proof whether microbes colonizing different plastics are distinct from each other and from other inert hard substrates, the bacterial communities of these different substrates were analysed using 16S rRNA gene tag sequencing. Our findings indicate that tightly attached microorganisms account to the rare biosphere and suggest the presence of plastic “*specific*” microorganisms/assemblages which could benefit from the given plastic properties or at least grow under limited carbon resources.

## Introduction

Since the middle of last century the increase of global plastics production is accompanied by an accumulation of plastic litter in the marine environment [1, 2]. Persistent plastic items are rarely degraded but become fragmented over time and are dispersed by currents and wind [1, 3, 4]. Consequently, marine plastic litter can be found in marine waters all over the globe.

In contrast to interactions of larger organisms with plastics, which are mainly characterised by the consequences of ingestion or entanglement, the interaction of microorganisms and plastics are of completely different nature. Plastics function as habitats and are rapidly colonized by marine microorganisms which form dense biofilms on the plastic surface, the so called “Plastisphere” [5]. Therefore, plastic litter is a substrate which can serve as a vector for the widespread distribution of a variety of organisms, including harmful algae species, barnacles, bryozoans [6, 7] as well as potentially pathogenic *Vibrio* species [5, 8]. The persistence of plastics in marine environments is a matter for debate, and estimates range from hundreds to thousands of years depending on the chemico-physical properties of the plastic type [4]. “Biofouling refers to the undesirable accumulation of a biotic deposit on a surface” [9] and can play a major role in controlling plastic buoyancy [10]. Additionally, biofouling also lead to deterioration resulting in fragmentation of larger plastic items and may also result in degradation of the polymers [11, 12].

Based on culture-independent approaches, the current state of knowledge regarding the “Plastisphere” is as follows; microbial communities on marine plastic debris differ consistently from the surrounding seawater communities [5, 13–15], the plastics community composition is driven by spatial and seasonal effects [13], the community composition varies with the substrate type [15, 16], and plastics biofilm composition is dependent on the habitational conditions, e.g. harbour vs. offshore [17]. Overall, the composition of marine plastics biofilms is probably resulting from a unique interaction of various factors such as the substrate type, the surrounding environment, the geographical location and the seasonal variation of environmental parameters. However, it is well established that several prokaryotic families build the general plastic biofilm community. These include *Flavobacteriaceae*, *Erythrobacteraceae*, *Hyphomonadaceae* and *Rhodobacteraceae* found in the North Sea, the coastal Baltic Sea, multiple locations in the North Atlantic, and freshwater systems [5, 17, 18].

Recent studies investigated the specificities of plastics communities comparing different types of plastics with other substrates such as wood or glass [14, 16, 18, 19]. Comparing the PET and glass associated microbiome, Oberbeckmann et al. [14] could not detect significant differences in community composition after 5 to 6 weeks of incubation. In contrast, Kirstein et al. [16] found significant differences between the community composition associated to diverse plastics and glass investigating mature biofilms (15 month). However, the differences in community composition were generally low, indicating that the shared core of the various biofilms is rather substrate unspecific. Furthermore, the strongest contribution to the total dissimilarity between the diverse substrates was often given by less abundant operational taxonomic units (OTUs). All this points towards the importance of rather rare species in plastic associated marine biofilms [16]. Considering that the competition pressure in mature biofilms can be particularly high (e.g. for space or nutrients), uncovering those rare species is a necessary first step to identify microbes that are closely associated/interact with the polymeric surface, which will select for species able to survive better when the competition pressure decreases.

To date, researchers of the “Plastisphere” have discussed the potential of plastic “*specific*” organisms/assemblages to be involved in biodegradation [5, 13–15, 17, 18, 20, 21]. Here, a plastic “specific” organism/assemblage is discriminating a respective plastic type from another substrate type. Several microorganisms, including bacteria and fungi, were isolated from various environments and were reported to have a degradative effect on specific plastic types [22, 23]. Regarding assemblages, recently Syranidou and colleagues developed tailored micro-consortia suggesting that those are capable of degrading weathered polystyrene (PS) and polyethylene (PE) fragments, respectively [24, 25].

Microbes generally have the potential to degrade complex organic compounds in various environments. This is raising the question, why significant differences between diverse plastics and other inert substrates could not be detected comparing young marine biofilms [14, 18, 19] or were found to be generally low between mature marine glass and diverse plastic biofilms [16]. Kirstein et al. [16] has evidence for a general marine biofilm core community of abundant bacterial taxa, which serve as shared core among diverse substrates, indicating that plastic “specific” microorganism might be represented by rather rare species. Assuming that these specificities of plastic biofilms are referring to microbes of the rather rare biosphere and that plastic “*specific*” microorganisms are closely attached to the polymeric surface; a three phase stepwise uncovering experiment was conducted. In Phase 1, nine distinct plastic films and glass as control were incubated in situ for 21 months in a natural seawater flow-through system. In Phase 2, a high-pressure water jet treatment technique was applied to remove the upper loosely attached biofilm layers, to unveil potential plastic “*specific*” microorganisms. Thereafter, in Phase 3, those treated films were used as a source for colonisation of the same type of sterile plastic strips. Illumina sequencing of the hypervariable V3/V4 region of the 16S rRNA gene was applied to analyse and compare the prokaryotic communities attached to the various substrates. In addition attached cells were visualized via Scanning Electron microscopy.

## Materials and methods

### Biofilm formation

**Fig 1:**
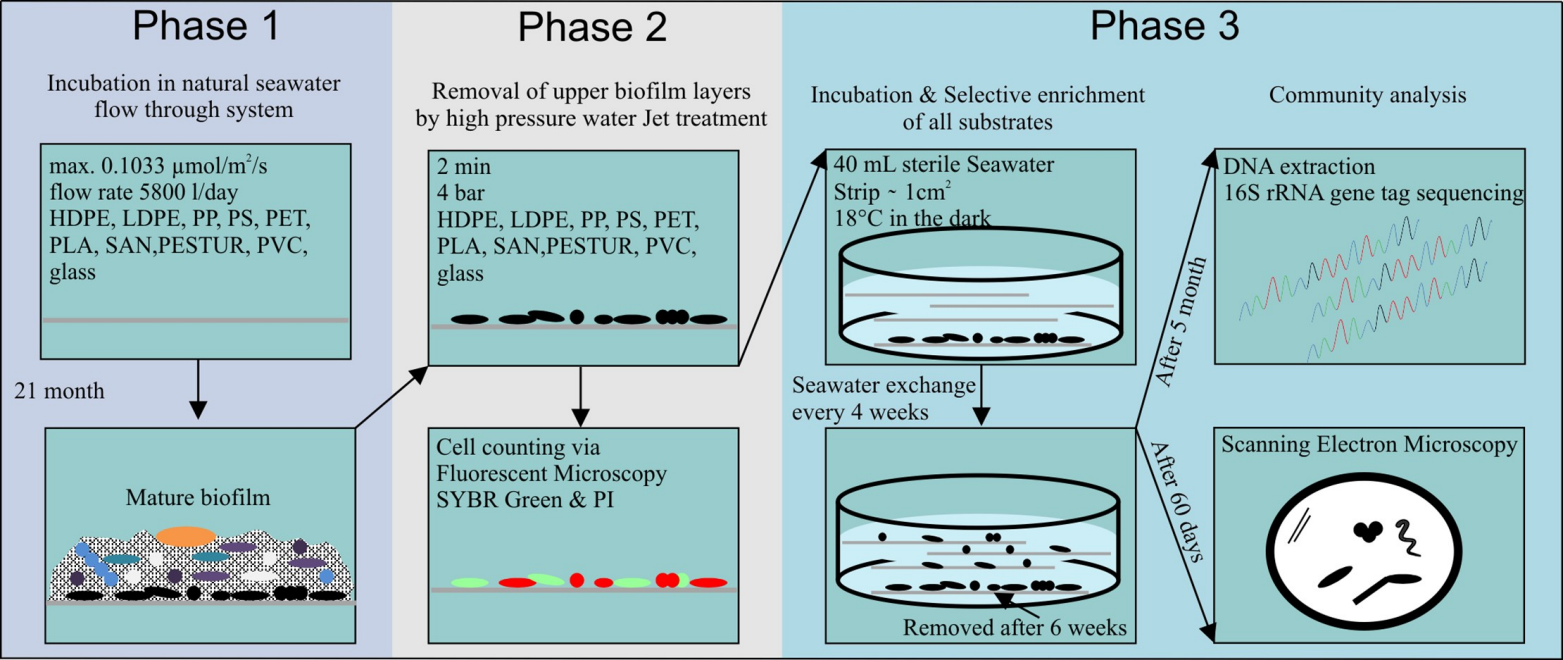
Experimental design. Schematic presentation of the three phased stepwise uncovering experiment of potential plastic “*specific*” bacteria.

A three phased experiment to stepwise uncover closely attached rare microbes was conducted (Fig 1). In Phase 1, biofilm formation was performed on 9 distinct plastic substrates such as high-density polyethylene (HDPE) (ORBITA-FILM GmbH), low-density polyethylene (LDPE) (ORBITA-FILM GmbH), polypropylene (PP) (ORBITA-FILM GmbH), polystyrene (PS) (Ergo.fol norflex GmbH), polyethylene- terephthalate (PET) (Mitsubishi Polyester Film), polylactic acid (PLA) (Folienwerk Wolfen GmbH), styrene-acrylonitryle (SAN) (Ergo.fol norflex GmbH), polyurethane prepolymer (PESTUR) (Bayer), polyvinyl chloride (PVC) (Leitz) (S1 Table). The term substrate refers here, to a surface on which an organism grows or is attached and which might serve as a carbon source. These substrates, highly abundant in the marine environment and on glass slides as a neutral control for 21 month in the dark (max. light intensity 0.1033 μmol/m^2^/s) in a natural seawater flow-through system located at the “Biologische Anstalt Helgoland” approximately 60 km off the German coastline. North Seawater was directly pumped through the system (flow rate of approx. 5800 l/day).

### Removal of the “upper” biofilm layers by high pressure treatment

**Fig 2:**
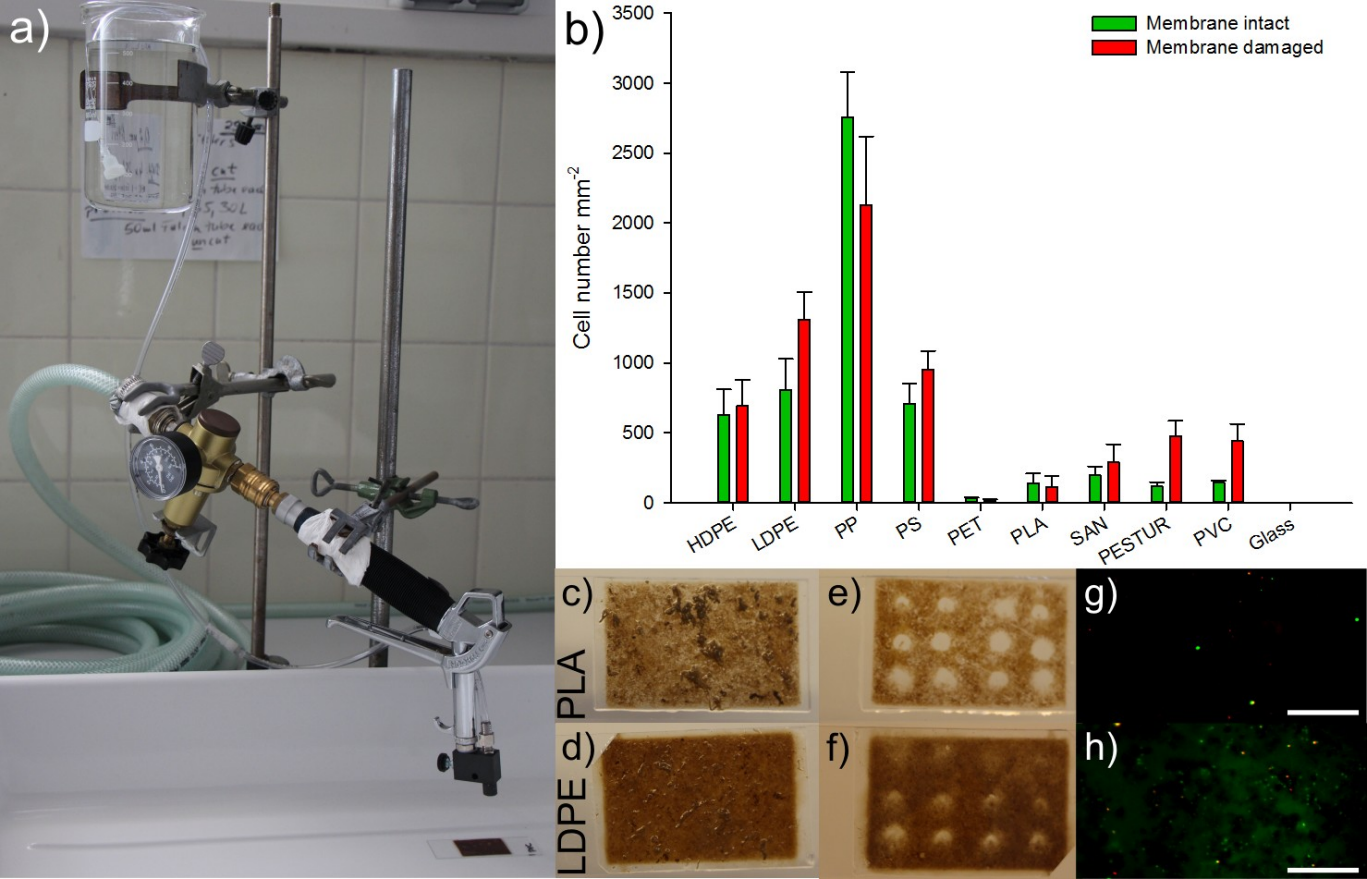
High-pressure water Jet treatment with the a) high pressure treatment device. b) Barplot of the enumerated mean of adherent membrane intact (green) and membrane damaged (red) cells after a high pressure treatment at 4 bar for 2 minutes, vertical bars denote the Standard Error. Photograph of the 21 month old biofilm attached to c) Polylactic acid and d) Low density polyethylene. Resulting spots e; f) in respective biofilms after high pressure treatment. Double stained (SYBR Green & PI) cells on respective substrate g; h) after high pressure treatment with, scale bars are 10 μm.

In order to remove the upper biofilm layers in Phase 2 of our stepwise experiment (Fig 1), a high-pressure treatment technique was developed to remove the loosely attached biofilm layers. This was performed with a mini high-pressure cleaning device (Lico-Tec; Arnstorf, Germany) established to shot (Fig 2A) sterile seawater (0.2 μm filtered and autoclaved) vertically onto the biofilm associated to the different substrates. Seawater was shot with a working distance of 1 cm for 2 minutes at 4 bar. Next, to evaluate and compare how many cells were still attached on each substrate after the high-pressure treatment, cell counting of all samples was performed. Therein, staining with propidium iodide (PI) and SYBR Green allowed distinguishing between membrane intact and membrane damaged cells (Fig 2G and 2H). The treatment was repeated 9 times on each plastic foil with every sample in triplicates. Fluorescence microscopy was investigated with the optical microscope Axioplan2, imaging (Zeiss; Oberkochen, Germany). Detection of the total cell number stained with fluorescent dye SYBR Green was performed with the filter set 09 (Zeiss; Oberkochen, Germany). To evaluate the proportion of damaged cells, the filter set 20 has been applied (Zeiss; Oberkochen, Germany). Detailed information on the development of the high-pressure treatment technique, staining, and visualization can be found in the supplement (S1 Appendix, S2 Table). ImageJ has been used for cell counting [26].

### Selective enrichment on distinct plastics

In order to enrich the uncovered potential plastic “*specific*” microorganisms a re-colonization experiment was designed. Therefore a strip of ~ 1 cm^2^ with associated 21 month old biofilm of each of the substrates was treated for 2 minutes at 4 bars with the high-pressure device by moving the strip slowly under the stream. These strips with the remaining closely surface attached microorganisms were transferred into sterile glass Petri dishes with 40 ml sterile filtered and autoclaved North Seawater. For each of the nine plastic types and glass, new ethanol sterilised strips of the same size were added to these Petri dishes and incubated at 18°C in the dark. All different substrate strips were sterilized in 70% ethanol and air dried before being placed in the Petri dishes. After six weeks the re-colonization source was removed (short-term). Due to manageable sample numbers only the second stage of experiments in Phase 3, the long-term incubation was carried out in replicates. Fresh sterile seawater was provided every four weeks. After 60 days one strip of each substrate was taken for visualization via SEM. After five months of incubation five replicates of each long-term incubated substrate were taken for DNA extraction followed by 16S gene tag sequencing.

### Scanning electron microscopy

Scanning electron microscopy was used to visualize the colonized plastics. Strips of each re-colonized substrate of about 0.5 cm^2^ with the attached cells were fixed at 4°C in sterile seawater containing 2.5% glutaraldehyde and 50 mM sodium cacodylate (pH 7.2). Samples were stored in the fixative at 4°C (4–10 days) until processing for scanning electron microscopy. The samples were stepwise dehydrated with ethanol bath series of 10 min each at concentrations of 30%, 50%, 70%, 90%, followed by 3 baths of 10 min in 100% ethanol. Samples were immediately critical point dried (BAL-TEC CPD 030). All samples were sputter coated (BAL-TEC SCD 005) with gold-palladium before observing with a field emission scanning electron microscope (JEOL JSM-7500F) with the in-lens detector (SEI-detector) at 5kV and a working distance of 8 mm [16].

### DNA extraction & 16S Illumina tag sequencing

After five month of selective enrichment, the DNA of microbial biofilms of the nine different short- and long-term incubated substrates was extracted using the PowerBiofilm DNA Isolation Kit (MOBIO Laboratories, Carlsbad, CA) according to the manufacturer's protocol, including mechanical pulping (FastPrep FP 120, ThermoSavant,Qbiogene, United States) for 40 seconds on level 4.0. DNA quantity was determined photometrically with a PicoGreen assay (Invitrogen, Waltham, MA) in duplicates using a Tecan Infinite M200 NanoQuant microplate reader (Tecan, Switzerland).

16S rRNA gene tag sequencing of the V3 / V4 fragment of the 16S rRNA was performed at LGC Genomics GmbH (Berlin, Germany). DNA fragments were amplified using amplification primers 341F (5’-CCTACGGGNGGCWGCAG-3’) and 785R (5’-GACTACHVGGGTATCTAATCC-3’) [27]. Primers also contained the Illumina sequencing adapter sequence and a unique barcode index. Resulting amplicons were paired-end sequenced 2 x 300 bp on an Illumina MiSeq platform. Paired-end reads were merged using BBMerge 34.48 software (http://bbmap.sourceforge.net/) and processed through the SILVAngs pipeline [28]. Sequences were de-replicated at 100% identity and further clustered with 98% sequence identity to each other. Representative sequences from operational taxonomic unit clusters (OTUs) were classified up to genus level against the SILVA v128 database using BLAST as first described by Ionescu et al. [29]. Sequences having an average BLAST alignment coverage and alignment identity of less than 93% were considered as unclassified and assigned to the virtual taxonomical group “No Relative" [28]. Finally, 1,307,882 (99.77%) classified sequences were obtained. For following downstream analyses, classifications on the genus-level were used to generate the final abundance matrixes. All classifications contained the sum of all sequences represented by OTUs with the equal taxonomic path. The raw sequence data is available in the European Nucleotide Archive [30] under the accession number PRJEB30284, using the data brokerage service of the German Federation for Biological Data [31], in compliance with the Minimal Information about any (X) Sequence (MIxS) standard [32].

### Statistics and downstream data analysis

To see whether the data of the cell counts were normally distributed R statistical software with the nlme package has been used. Generalized linear models (GLM) were used to explain the variability of the attached cells during the establishment of the final LicoJet treatment as well as in the viability assay. GLM are used in statistics to generalize linear regression with variables that have an error distribution not normally distributed [33].

Species richness (S) of the bacterial communities on different short- and long-term incubated substrates was calculated based on read counts of operational taxonomic units (OTUs).

For beta diversity analysis, first the virtual taxonomical group “No Relative” was removed from further analysis. Next, counts per classification were normalized by calculating their relative abundances to the total number of SSU rRNA gene reads per sample. OTUs with a minimal mean relative abundance of less than 0.1% in at least one substrate type were excluded.

Permutational multivariate analysis of variance (PERMANOVA) was used to test for statistically significant variance among the source and re-colonized communities attached to the different substrates. PERMANOVA was carried out with fixed factors and 9999 permutations at a significance level of p < 0.05. Homogeneity of dispersion (PERMDISP) was applied, to test whether data in significant PERMANOVA results were not over dispersed, using 9999 permutations at a significance level of p < 0.05. To visualize patterns of samples regarding various substrates, source and re-colonized communities, principal coordinates analysis (PCO) using Hellinger distance (D17; [34]) was performed.

To determine OTUs that discriminated the various re-colonized substrates from each other similarity percentage analysis (SIMPER) was applied. SIMPER was performed using Bray Curtis similarity (S17) with fourth root transformed relative abundances.

For shade plot creation of unveiled plastic “*specific*” taxa, first all OTUs with a mean relative abundance of at least 0.1% present on both, plastics and glass, were rejected. Next, OTUs contributing most (> 3%) to the total dissimilarity between different plastic groups (SIMPER analysis) were subjected into cluster analysis. This trimmed data set resulted in 23 OTUs to that the moderate square root transformation was applied. To determine which groups of plastics cluster together in respect of plastic “*specific*” taxa, hierarchical cluster analysis was performed using Bray Curtis similarity (S17) using square root transformed relative abundances. To test our hypothesis that specificities of plastics biofilms might be related to members of the rather rare biosphere the plastic “*specific*” OTUs were compared with a former dataset of 15 month old biofilms origin of the same experimental set up (Sequence data deposited in the European Nucleotide Archive under the accession number PRJEB22051).

Alpha diversity, PERMANOVA, PERMDISP, PCO, SIMPER and CLUSTER analysis were carried out with the Primer 7 software package plus the add-on package PERMANOVA+ (PRIMER-E Ltd, UK).

## Results

### Evaluation of adherent cells

Cell counts revealed that after the high-pressure treatment both, cells with intact and damaged membranes were still attached to the different plastics (Fig 2B). A total cell count of the adhesive cells on each substrate revealed that attachment occurred to the largest extent on PP followed in the range of LDPE, PS, HDPE, PESTUR, PVC, SAN, PLA, PET and at least on glass. Furthermore it is noticeable that mostly the mean of membrane damaged cells exceed the mean membrane intact cells except for PP, PET and PLA. The mean cell numbers of PP by far outnumbered the cell counts of all other substrates (Fig 2B). Both states, of membrane damaged and intact cells were significantly dependent on the substrates (S3 Table).

### Scanning electron microscopy of colonized plastics

**Fig 3:**
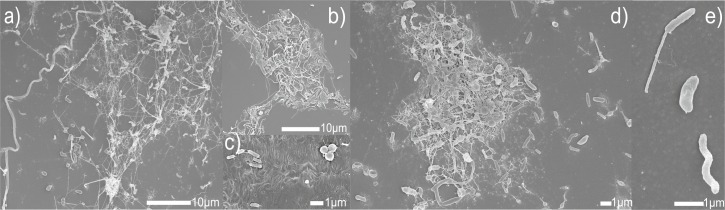
SEM images of colonized plastics. a) Meshwork of morphological diverse cells embedded in EPS attached to PS. b) Colony attached to PESTUR c) Single cells of rods and cocci on HDPE d) Consortia of rods and cocci embedded in EPS on PS e) Rod with spore, comma and spiral cells on PVC.

To prove successful colonization, after 60 days of incubation in sterile seawater, plastic strips were visualized by SEM (Fig 3). Examination of the plastic strips by SEM confirmed re-colonization of all substrates and provided a closer picture of the microbes attached to the diverse substrate surfaces (Fig 3).

Various microbial species of different morphologies connected through a network of EPS or solely distributed across the surface without visible adhesive structures were observed (Fig 3). Exemplarily, Fig 3A, 3B and 3D are showing morphological diverse bacteria embedded in EPS building colonies on the polymeric surfaces of PS and PESTUR. Fig 3C) shows rods and cocci attached to HDPE and on Fig 3E) three single cells of different morphologies present on PVC are shown.

### Selective enrichment & community analysis

**Fig 4:**
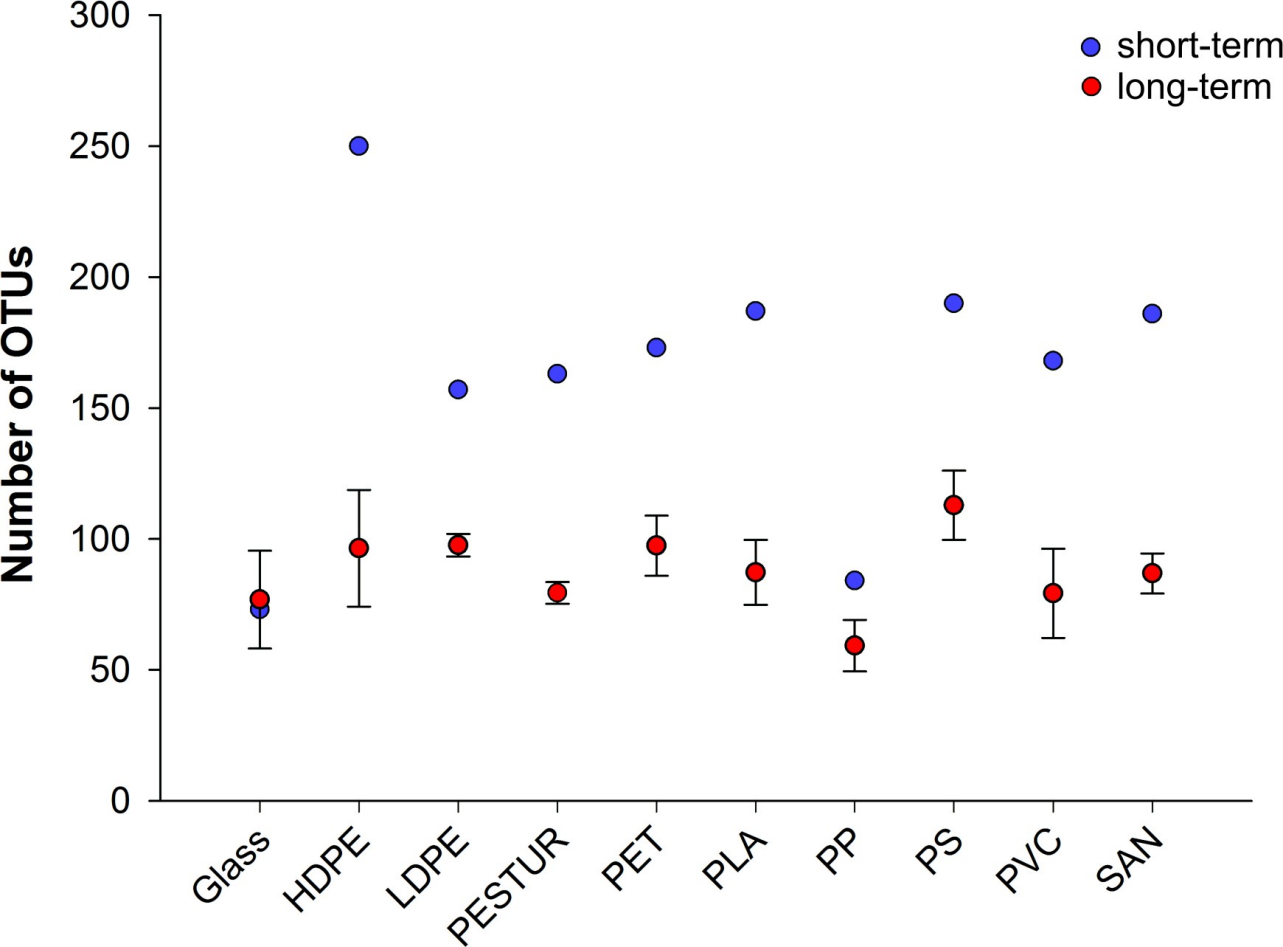
Richness of the bacterial communities attached to the diverse substrates based on the number of observed OTUs. Vertical bars denote the standard deviation (n_short-term_ = 1; n_long-term_ = 5).

For selective enrichment the high-pressure treated plastics (comprising the attached source community) were incubated with newly provided strips of the same polymer kind. The source community strips were removed after six weeks (short-term) of incubation and after further five month (long-term) of selective enrichment the taxonomic composition of the bacterial communities on the diverse substrates were analysed in detail by 16S rRNA gene tag sequencing. The species richness of the different samples, analysed by calculating the number of observed OTUs (number of species (S)) and Margalef`s species richness (d) (Fig 4, S6 Table), showed that the short-term communities had a higher richness compared to the long-term communities on all substrates but glass (Fig 4), what point towards a selection of “specific” microbes on the respective plastic type.

**Fig 5:**
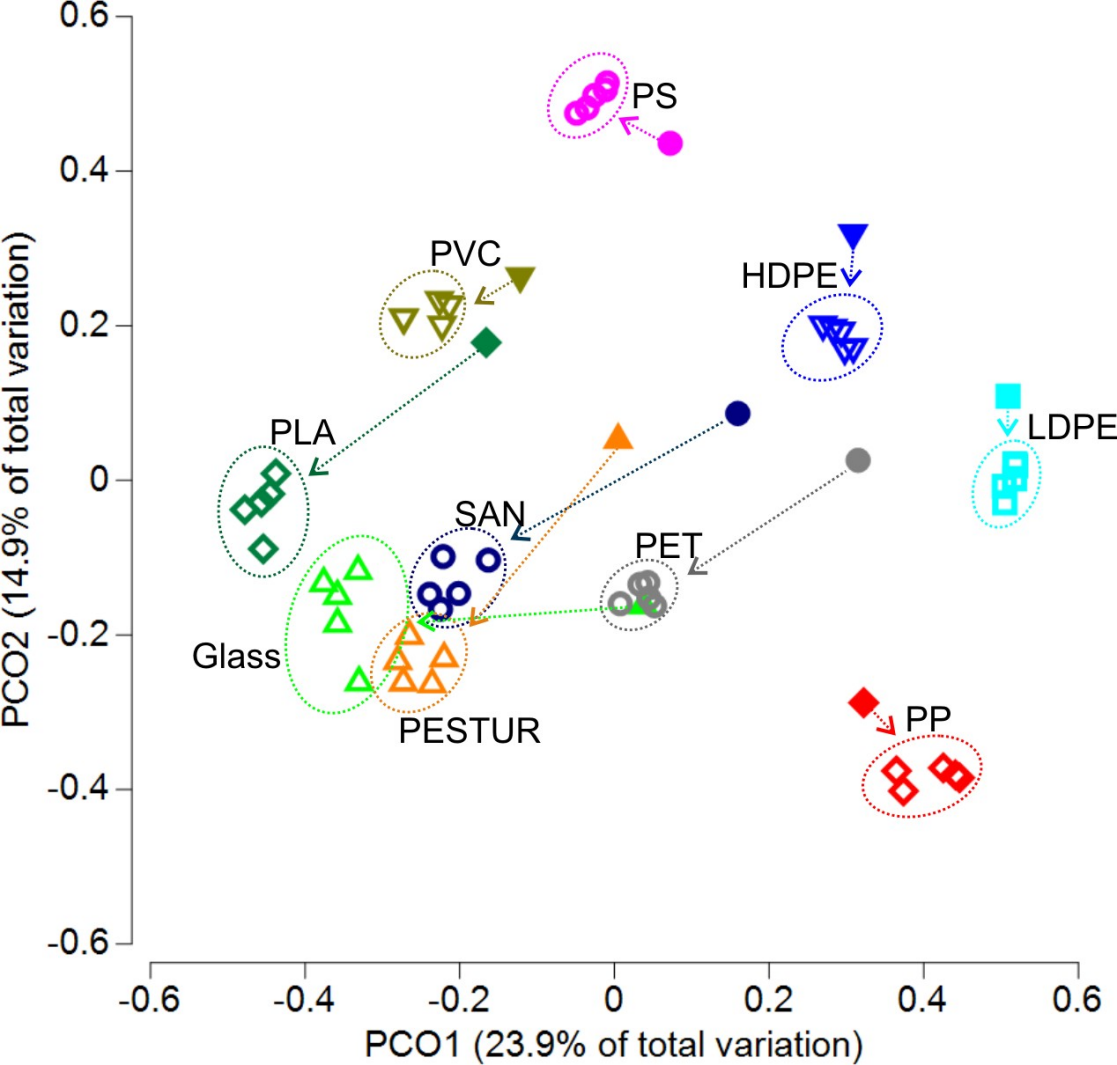
Principle Coordinate Ordination relating variation in the community composition between different short- and long-term incubated substrates. PCOs representing similarity of biofilm communities based on relative abundances of OTUs across samples. OTUs with a mean relative abundance of at least 0.1% in one substrate type (n_short_ = 1; n_long_ = 5) were analysed. The different colours indicate the respective substrate, filled symbols represent short-term samples, open symbols long-term samples. Arrows connect short- and long-term samples of the respective substrate.

Principle coordinate analysis was used to visualize the similarities and dissimilarities between the various short- and long-term communities (Fig 5). First, all samples of all substrates were clearly divided (Fig 5). Second, the short-term communities of HDPE, LDPE, PP, PS and PVC clustered nearby their related long-term communities whereas the short-term community of glass, PLA, PESTUR, SAN and PET clustered more distant to their long-term communities (Fig 5). However, the first two axes merely represent 38.8% of the total variation within the analysed communities. PERMANOVA analysis confirmed that the selective enriched long-term communities differed significantly between all colonized substrate types (*p*<0.05; pairwise PERMANOVA, S5 Table).

**Fig 6:**
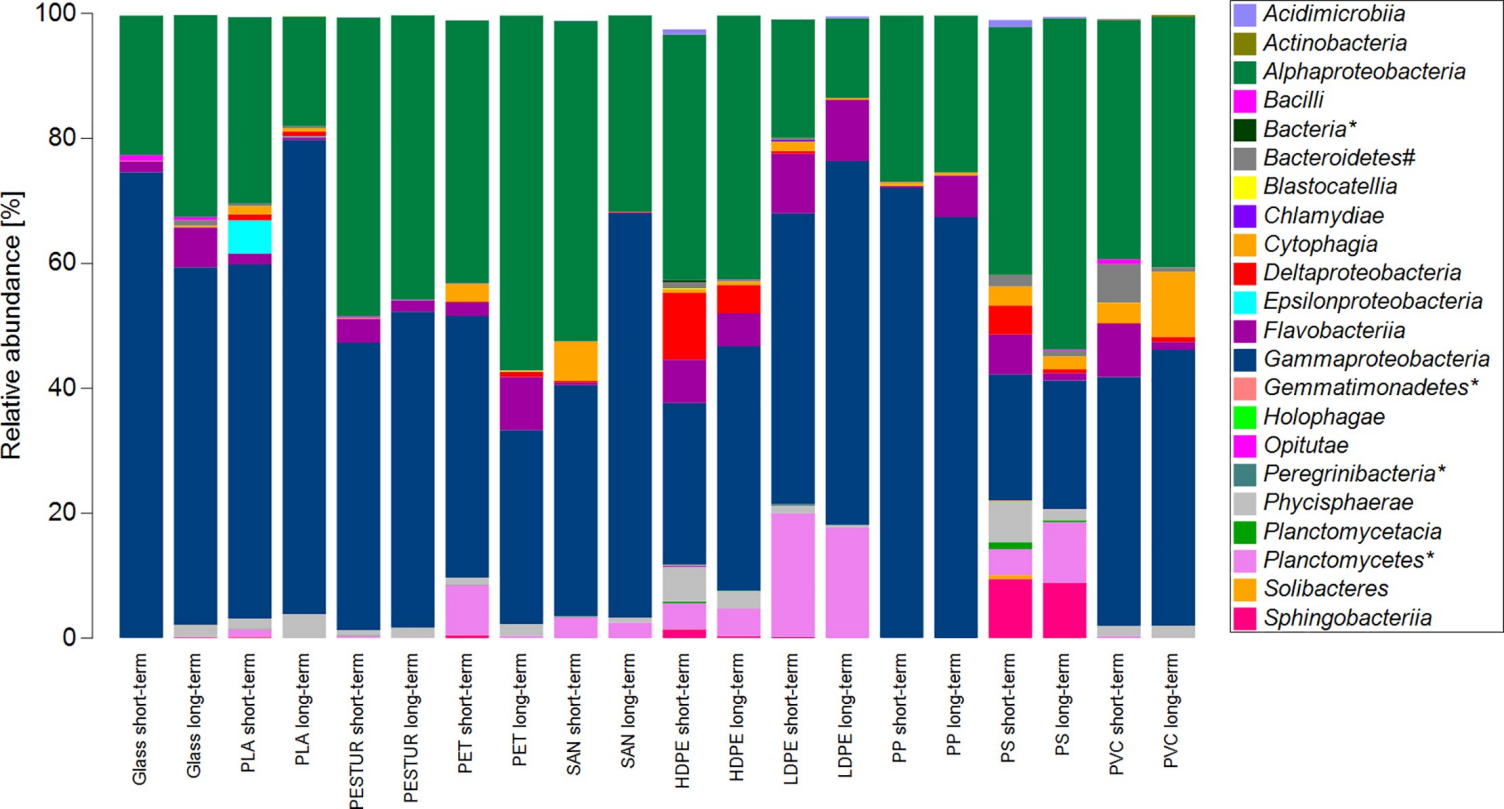
Biofilm community composition based on abundance profiles of the short- and long-term communities on the class level on different plastics and glass. OTUs with a mean relative abundance of at least 0.1% in one substrate type (n_short_ = 1; n_long_ = 5) were analysed. A * indicates the term “unclassified”, a # indicates the term “*Incertae Sedis*”.

The bacterial community of short- and respective long-term incubated substrates displayed a change in community composition during the time of selective enrichment. Overall, *Alpha*- (18–53%) and *Gammaproteobacteria* (20–75%) displayed the highest relative abundances in all samples of all substrates (Fig 6). Some classes were abundant in the short-term communities but nearly disappeared over the time of selective enrichment e.g. the class of *Epsilonproteobacteria* on PLA or *Cytophagia* on SAN (Fig 6). Vice versa, some classes showed lower abundances in the short- than in the long-term samples e.g. *Flavobacteria* on PP, PET and glass. The class of *Sphingobacteria* appear to be characteristic for PS as this class was nearly equally abundant in the short- and in the long-term samples (Fig 6).

### Uncovered plastic “*specific*” bacteria

**Fig 7:**
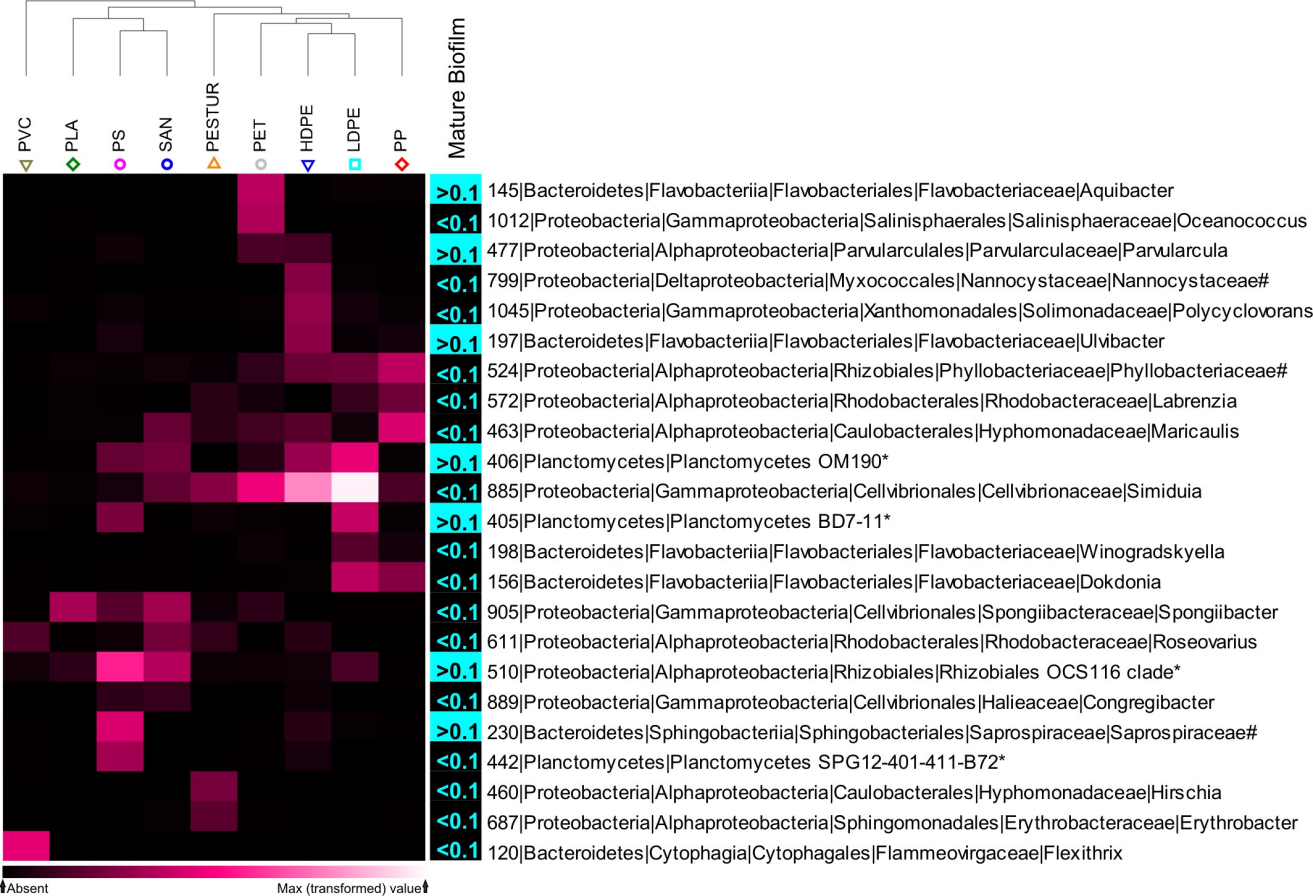
Shade Plot of plastic “*specific*” OTUs (indicated by numbers) on different long-term plastics and comparison of their relative abundance in untreated mature biofilms of the same experimental set up after 15 month. Abundant OTUs (mean relative abundance <0.1%; n = 50) are indicated in turquoise, rather rare OTUs (mean relative abundance >0.1%; n = 50) are indicated in black. Shade Plot creation was based on square root transformed relative abundances. OTUs with a mean relative abundance of at least 0.1% in one substrate type (n = 5) were analysed. Displayed are OTUs jointly contributing, with a minimum of 3%, to the total dissimilarity between different plastic groups (SIMPER analysis). OTUs with a mean relative abundance of at least 0.1% present on both, plastics and glass, were rejected. The amount of contribution is indicated by the colour of cells, lighter colours represent higher contributions. A * indicates the term “unclassified”, # indicates the term “uncultured”.

Long-term enriched communities associated with different substrates differed between 35–66% from each other (S7 Table). For hierarchical clustering OTUs with a mean relative abundance of at least 0.1% present on both, plastics and glass were rejected, resulting in 68 OTUs (S3 Fig). To visualize patterns of mostly discriminating members, OTUs jointly contributing with a minimum of 3% (max. dissimilarity between plastics = 6.07%), to the total dissimilarity between different plastic groups (SIMPER analysis) were subjected into cluster analysis. Accordingly, the trimmed data set resulted in 23 mostly discriminating and therefore potential plastic “*specific*” OTUs (Fig 7). The hierarchical clustering of the potential plastic “*specific*” OTUs indicated closest relatedness of HDPE and LDPE (polyolefins) as well as of PS and SAN (styrenes), whereas e.g. PVC cluster clearly away from all other plastics (Fig 7). This differences or similarities are caused by the presence or absence of particular OTUs, or related to differences in relative abundances of OTUs in common. The main reason for the distinctness of PVC is an OTU assigned to the genus *Flexithrix*, with relative abundances of >5% on PVC (Fig 7 and S3 Fig). The genus *Hirschia* and *Erythrobacter* contributed to the dissimilarity between PESTUR and all other plastics (Fig 7 and S3 Fig). Whereas an OUT assigned to the uncultured *Phyllobacteriaceae* contributed to the similarity between the polyolefins HDPE, LDPE and PP.

Comparison of the resulting 23 OTUs with a former dataset of 15 month old biofilms attached to the same substrates [16] revealed that 16 out of the 23 OTUs related to the rather rare biosphere (relative abundance <0.1%) including *Oceanococcus* (OUT 1112), *Nannocystaceae* (OUT 799), *Polycyclovorans* (OUT 1045), *Phyllobactereacea* (OUT 524), *Labrenzia* (OUT 572), *Maricaulis* (OUT 463), *Simiduia* (OUT 885), *Winogradskyella* (OUT 198), *Dokdonia* (OUT 156), *Spongiibacter* (OUT 905), *Roseovarius* (OUT 611), *Congregibacter* (OUT 889), *Planctomycetes* SPG12-401-411-B72 (OUT 442), *Hirschia* (OUT 460), *Erythrobacter* (OUT 687) and *Flexithrix* (OUT 120). Seven OTUs assigned to *Aquibacter* (OUT 145), *Ulvibacter* (OUT 197), *Planctomycetes* OM190 (OUT 406), *Planctomycetes* BD7-11 (OUT 405), *Parvularcula* (OUT 477) *Saprospiraceae* (OUT 230) and Rhizobiales OCS 116 (OUT 510) showed relative abundances >0.1% in the mature biofilms (Fig 7).

## Discussion

Identification of microbes that preferentially colonize and interact with plastics surfaces remains challenging as the differences in community composition of mature biofilms are generally low [16]. Furthermore, young biofilms (2–6 weeks) appear to be rather unspecific between different plastic types or other inert substrates like glass [14, 18, 19]. Here, we present a three phase experimental approach to uncover potential plastic “*specific*” microbes. Our findings indicate that tightly attached microorganisms might account to the rather rare biosphere and suggest the presence of plastic “specific” microorganisms/assemblages which could possibly benefit from the given plastic properties.

### Water jet treatment & selective enrichment

As the main hypothesis of this study was that plastic “*specific*” microorganisms are tightly attached to the polymeric surface, a technique to remove the upper loosely attached part is the first step to facilitate further analysis. There are numerous studies trying to achieve a complete sanitation of biofilms but, to the best of our knowledge, no method can successfully achieve entire detachment [35]. The persistency of biofilms towards removal techniques, as inauspiciously as it may be for sanitation issues, is of great advantage to investigate these strongly adherent cells on the substrate. Techniques to remove the cohesive layers of the biofilm, while leaving the adhesive layer attached to the substrate on purpose, are not published. Since chemical or enzymatic action can break adherent bonds, removal of the coherent biofilm layers requires mechanical action which does not seem to have much influence on the biofilms integrity [36, 37]. Microscopic investigations revealed that strongly attached microbes were able to survive the high-pressure water Jet treatment on all plastics with the largest extent of adhesive cells on PP followed by LDPE, PS, HDPE, PESTUR, PVC, SAN, PLA, PET and at least on glass. Already in 1979, Fletcher and Loeb [38] examined substrates with a hydrophilic and positive to neutral surface charge, revealing a moderate number of cells, while only very few cells stayed attached to hydrophilic and negatively charged surfaces such as glass. This might explain the variation in cell numbers between the diverse substrates as well as the low cell numbers found on glass compared to those on the nine different plastic types after the high pressure removal in this study.

Differentiated communities (short-term *vs*. long-term) developed within the third phase of the experiment after five month under nutrient limited conditions in the sterile seawater incubation. PERMANOVA pairwise comparison indicated that all microbial communities on their respective substrate differed significantly to each other. Although all substrates were treated similarly, it should be noted that differences in community profiles could be induced by the considerable difference in remaining cell numbers on diverse substrates after the high-pressure water Jet treatment. However, the detected differences still imply that the substrate shaped the community as a result of the adherence strength of the biofilm to the respective substrate surface. Comparing short-term (six weeks) and long-term (five month) incubated communities revealed shifts towards communities with lower richness over time for all plastic types but glass, which points towards a selection of microbes, that are either specialised to low nutrient conditions or the respective plastic type. On the class level, three different changes were observed between short- and long-term incubated communities; a shift from high to low abundant classes, and vice versa, but also classes being characteristic for a plastic type, implying that the plastic type is responsible for shaping the community composition. Biofilm communities include a heterogeneity in form of organisms with various metabolic capacities and different physiological properties which generates on the one hand competition but also provides on the other hand opportunities for cooperation [39]. Hence, some of the observed changes in community composition might be related to organisms playing a specific role in interspecies interactions (cooperation) in plastic-degrading microbial assemblages.

### Unveiled potentially plastic “*specific*” microbes

The three phases stepwise uncovering of potential plastic “*specific*” bacteria resulted in 23 final OTUs contributing highly to the total dissimilarity between the nine plastic types. Generally, the chemical composition (e.g. polyesters, polyolefines) and physico-chemical properties of different plastic types, including the ones used in this study, are highly diverse in order to meet the different needs of thousands of end products [40]. The plastic foils used as substrate in the present study, are commonly produced for e.g. packaging and construction. It was hypothesized that plastic “*specific*” microorganisms are tightly attached to the polymeric surface and might be represented by rare but active species, since differences of mature biofilms between distinct plastic types were found to be generally low [16]. Comparing our two datasets revealed that 70% of the uncovered potential plastic “*specific*” OTUs of the present study, were assigned to the rather rare biosphere (<0.1%) of the biofilms investigated six month earlier (15 month old biofilm [16]). Former research reports that rare phylotypes tend to stay rare [41, 42]. Other studies suggested that rare but active populations might be controlled by top-down forces (e.g. predation) or competition (e.g. space, nutrients) within the biofilm and to underlie environmental controls (e.g. temperature) [43]. Having the potential to increase in abundance [44], our findings clearly support the idea that potential plastic “*specific*” species are, at least partly, controlled by competitive interactions in mature dense biofilms.

Several studies have investigated microbial communities on marine plastics under various conditions [5, 13, 14, 17, 18, 20, 21, 45]. After subtracting OTUs also abundant on glass, in total 68 OTUs were found specifically associated with the different plastics and, out of those, 23 mostly discriminating the chemically distinct plastics. Several researchers, reported about multiple families in common on a variety of marine plastics in different locations e.g. *Nannocystaceae*, *Flavobacteriaceae*, *Planctomycetes*, *Saprospiraceae*, *Erythrobacteraceae*, *Hyphomonadaceae* and *Rhodobacteraceae* [5, 14, 16–18, 21, 45]. Members of these families were also present within the 23 most discriminating OTUs in this study. Two OTUs were discriminating PESTUR from all other plastics assigned to *Hirschia* (*Hyphomonadaceae*) and *Erythrobacter* (*Erythrobacteraceae*). Several studies have previously reported about the abundance of these two families associated to diverse plastics in different experimental approaches and locations [5, 17, 18]. Recently, Oberbeckmann et al. [18] reported about the two families *Hyphomonadaceae* (mostly *Hyphomonas*) and *Erythrobacteraceae* (mostly *Erythrobacter*), being exclusively abundant in two weeks old biofilms on PE and PS. The genera *Erythrobacter* and *Parvularcula* were reported to be part of plastic biofilms in the North Atlantic and North Adriatic Sea [5, 45]. In our study one OTU belonging to the family *Saprospiraceae* was highly discriminating PS from the other plastics. Oberbeckmann et al. [18] also detected members of this family on diverse substrates, PE and PS just being one of them. *Phyllobacteriaceae* were found to be significantly more abundant on plastics, despite showing overall high relative abundances in the study of Oberbeckmann et al. [18]. In our study *Phyllobacteriaceae* contributed to the similarity between the polyolefins HDPE, LDPE and PP.

Since bacterial families are large, the uncovered plastic “*specific*” genera were compared with former studies and found four genera already recognized on marine plastics as *Roseovarius* [45], *Erythrobacter* [5, 18, 45] *Ulvibacter* [14] and *Parvularcula* [5, 45]. The repetitive detection of these genera associated to marine plastics in various approaches suggests that further investigations on their role in plastic biofilms are required. However, since our experimental design focused on the enrichment of tightly attached and rather rare taxa, they might have been present but not recognized in previous research. For example, in the study of Kirstein et al. [16] the genera *Roseovarius* and *Erythrobacter* accounted to the rare biosphere (<0.1%) in the mature biofilms (15 month) and were therefore further not considered. Interestingly, in other studies *Roseovarius* and *Erythrobacter* were detected in relatively young (2 weeks) or in biofilms of unknown age [5, 18, 45].

### Sensing the surface–plastic properties

Sensing of a non-soluble surface followed by the successful colonization are the first steps for marine bacteria to develop a community, potentially leading to plastic biodegradation [46, 47]. Beside surface properties like hydrophobicity and roughness, surface chemodynamics like surface conditioning or nutrient enrichment also play a role in forming distinct biofilm communities [47]. This questions whether we, and other researcher, were detecting *“plastic specific”* organisms or *“plastic specific coatings”* organisms needs to be addressed in future studies. In the present study, bacterial taxa able to survive on glass likely used dissolved organic carbon present in the sterile seawater as carbon source, and consequently did not benefit from plastics surface properties or chemical composition. All other OTUs detected on the various plastic types were therefore potentially plastic *“specific”*. Due to short read lengths of 16S rRNA gene tag sequencing, a conclusive identification on the species level of the unveiled plastic “*specific*” OTUs was not possible so far. Since successful surface colonisation does not prove a special role as e.g. plastic degradation, the next step must be the systematic isolation and identification of those plastic *“specific”* organisms and to further test for the potential of one species or consortium to degrade the respective plastic type. On the community level, the next steps should be the disclosure of the mechanisms that allow the plastic “specific” assemblages to survive, their possible metabolic pathways and enzymes involved.

## Conclusion

This study represents a systematic and robust experimental approach uncovering potential plastic “*specific*” microbes and is therefore a step forward in understanding the substrate specificity of the “Plastisphere”. For the first time, a high-pressure water Jet treatment technique was used to remove the cohesive layer of mature biofilms, while leaving the adhesive layer on the plastics surface. Our results indicate the presence of plastic “*specific*” microorganisms/assemblages which could possibly benefit from the given plastics properties. Furthermore, our findings clearly indicate that plastic “*specific*” microorganisms might account to the rather rare biosphere and are tightly surface attached. Underrepresentation, due to low read counts, might be an explanation why specificities between plastics biofilms in natural marine environments were not detected so far in young biofilms or seem to be generally low in mature biofilms.

## Supporting information

S1 Appendix Development of the new high-pressure treatment technique and Visualization of high-pressure treated biofilms. (PDF)

S1 Fig Barplot of the cell numbers per mm2 evaluated at different time and pressures. The bars represent the different pressures with 2, 3, 4 bar at 2, 3, 4 minutes respectively. The vertical bars denote the Standard Error of the data.
(PDF)

S2 Fig Abundance profiles of the source (short-term) and re-colonized (long-term) communities on the family level on different synthetic polymers and glass. OTUs with a mean relative abundance of at least 0.1% in one substrate type (nsource = 1; nre-col = 5) were analysed. Displayed are taxonomic families with abundances of > 1% in at least one substrate type. The group `others`was made up of families with abundances < 1%. A * indicates the term “unclassified”.
(PDF)

S3 Fig Discriminative OTUs of the nine different plastics (n = 5). OTUs with a mean relative abundance of at least 0.1% (n = 5) in at least one substrate type were analysed. Displayed are OTUs jointly contributing to the total dissimilarity of at least 3% between plastic or with relative abundance of at least 1% on one substrate type. OTUs with a mean relative abundance of at least 0.1% present on both, plastics and glass, were rejected. The amount of contribution is indicated by the colour of cells, darker colours represent higher contributions. A * indicates the term “unclassified”, # indicates the term “uncultured”.
(PDF)

S1 Table Sample information about synthetic polymers used within this study. (XLSX)

S2 Table GLM model results of cell counts against exposure time and pressure. Both variables and their interaction resulted not significant (p-value > 0.05). Est. Average represents the estimated average, Std. Error represents the standard error.
(XLSX)

S3 Table GLM model results of cell count distinguished in membrane damaged and intact cells after a high pressure water treatment at 4 bars for 2 minutes and staining with PI and SYBR Green. Both variables and their interaction resulted significant (p-value < 0.05). Est. Average represents the estimated average of the mean cell counts, Std. Error represents the standard error of the mean cell counts.
(XLSX)

S4 Table PERMANOVA main tests of biofilm community on different re-colonized synthetic polymers and glass based on Hellinger distance of operational taxonomic units (OUTs). P-values were obtained using type III sums and 9999 permutations under the full model. d.f.: degrees of freedom, SS: sums of squares; MS: mean squares, perms: number of unique permutations per comparison. Significant results (p (perm) < 0.05) are highlighted in bold.
(XLSX)

S5 Table PERMANOVA and PERMDISP pair-wise tests biofilm communities on different re-colonized synthetic polymers and glass based on Hellinger distance of operational taxonomic units (OUTs). Significant results (p (perm) < 0.05) are highlighted in bold.
(XLSX)

S6 Table Univariate Diversity indices of biofilm communities on different re-colonized synthetic polymers and glass based on read counts of operational taxonomic units (OUTs). S: Total species, N: Total individuals, d: Species richness (Margalef), J': Pielou`s evenness, H'(log2): Shannon.
(XLSX)

S7 Table SIMPER analysis of re-colonized communities jointly contributing to the total similarity within and dissimilarity between different groups of synthetic polymers and glass. Av.Si%: average percentage similarity within the different groups, Av.δi%: average dissimilarity between the different groups.
(XLSX)
