# Supplementary material for: The Plastisphere – Uncovering tightly attached plastic “specific” microorganisms
Source: PLoS One. 2019 Apr 23;14(4):e0215859. doi: 10.1371/journal.pone.0215859 (PMC6478340; doi:10.1371/journal.pone.0215859)
Supplement: S1 Appendix — (PDF) [file pone.0215859.s001.pdf]

Development of the new high-pressure treatment technique

To develop the method and to evaluate whether there is a significance of time or pressure a pre-test with Polypropylene (PP) in triplicate has been performed (Figure S1). The high-pressure device (LicoJet) needs to be affiliated to a compressed air supply to create an air flow with high velocity through the device towards the restricted opening of the nozzle. The liquid inside the device nozzle gets pressed out by the pressurized air with the adjusted pressure. The LicoJet was held in a mounting structure to ensure time of spraying and nozzle distance to be controlled. Sterile seawater (0.2 µm filtered and autoclaved) was shot vertical, with a working distance of 1 cm on the biofilm associated to the different substrates in a time series of 2, 3 and 4 minutes, and a change in pressure at 2, 3 and 4 bar. The exposed spots were stained with SYBR Gold to determine the total cell count. Evaluation of the cell counts of remaining strongly attached cells on the substrate showed that neither the impacted pressure of the water current nor the duration of the pressure had any significant influence on the amount of cells (Figure S1, Table S2).

Visualization of high-pressure treated biofilms

To distinguish cells with membrane integrity from the ones with a damaged cell membrane after high-pressure treatment double staining with propidium iodide (PI) and SybrGreen was performed. In this study a mix of both stains was prepared according to the concentrations investigated by Falcioni et al (2008). In total, 20 µl of the double stain were added on each high-pressure treated spot and stained for 30 minutes at room temperature in the dark. After the staining process the polymeric foils were washed in deionized water to remove the unbound staining solution and dried with Whatman paper. To prevent the fluorescent from rapid photobleaching, the sample got fixed with a 0.1% (v/v) p-phenylenediamine anti-fade mounting medium. SybrGreen stained cells were detected with the optical microscope Axioplan2, imaging (Zeiss; Oberkochen, Germany) using a bandpass excitation filter with the wavelengths between 450 to 490 nm and a longpass emission filter of 515 nm into the IR spectra (filter set 09; Zeiss; Oberkochen, Germany). To evaluate how many cells of the total amount have a damaged cell membrane a bandpass excitation filter that passes light at a wavelength of 534 to 558 nm and is therefore ideal to excite PI has been used (filter set 20; Zeiss; Oberkochen, Germany). The emission filter passes the fluorescence from 575 to 640 nm and therefore transmits the emission of PI (617 nm) but excludes emission of SYBR Green (512 nm).
