# Supplementary figures and images for: The Plastisphere – Uncovering tightly attached plastic “specific” microorganisms
Source: PLoS One. 2019 Apr 23;14(4):e0215859. doi: 10.1371/journal.pone.0215859 (PMC6478340; doi:10.1371/journal.pone.0215859)

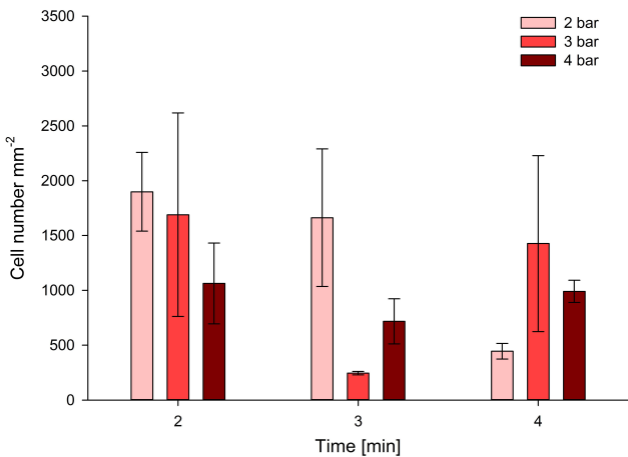

Supplement: S1 Fig — The bars represent the different pressures with 2, 3, 4 bar at 2, 3, 4 minutes respectively. The vertical bars denote the Standard Error of the data. (PDF) [file pone.0215859.s002.pdf]

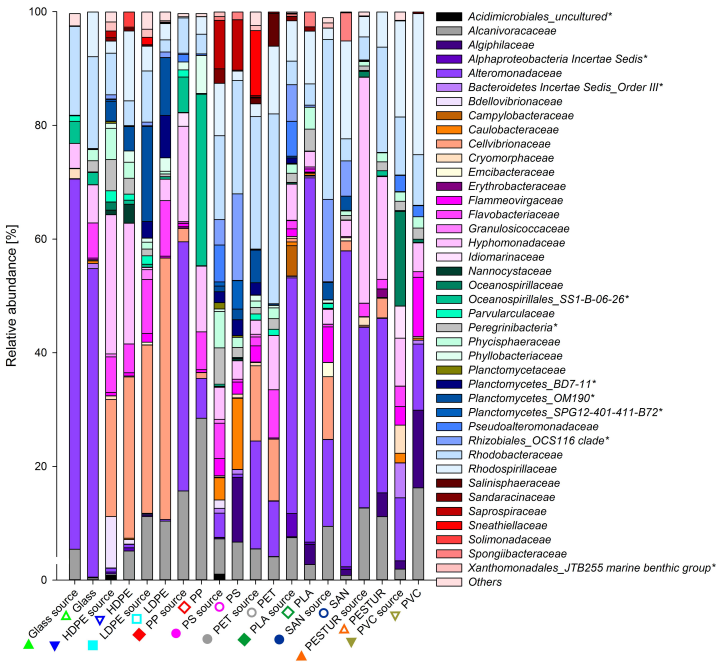

Supplement: S2 Fig — OTUs with a mean relative abundance of at least 0.1% in one substrate type (nsource = 1; nre-col = 5) were analysed. Displayed are taxonomic families with abundances of > 1% in at least one substrate type. The group `others`was made up of families with abundances < 1%. A * indicates the term “unclassified”. (PDF) [file pone.0215859.s003.pdf]

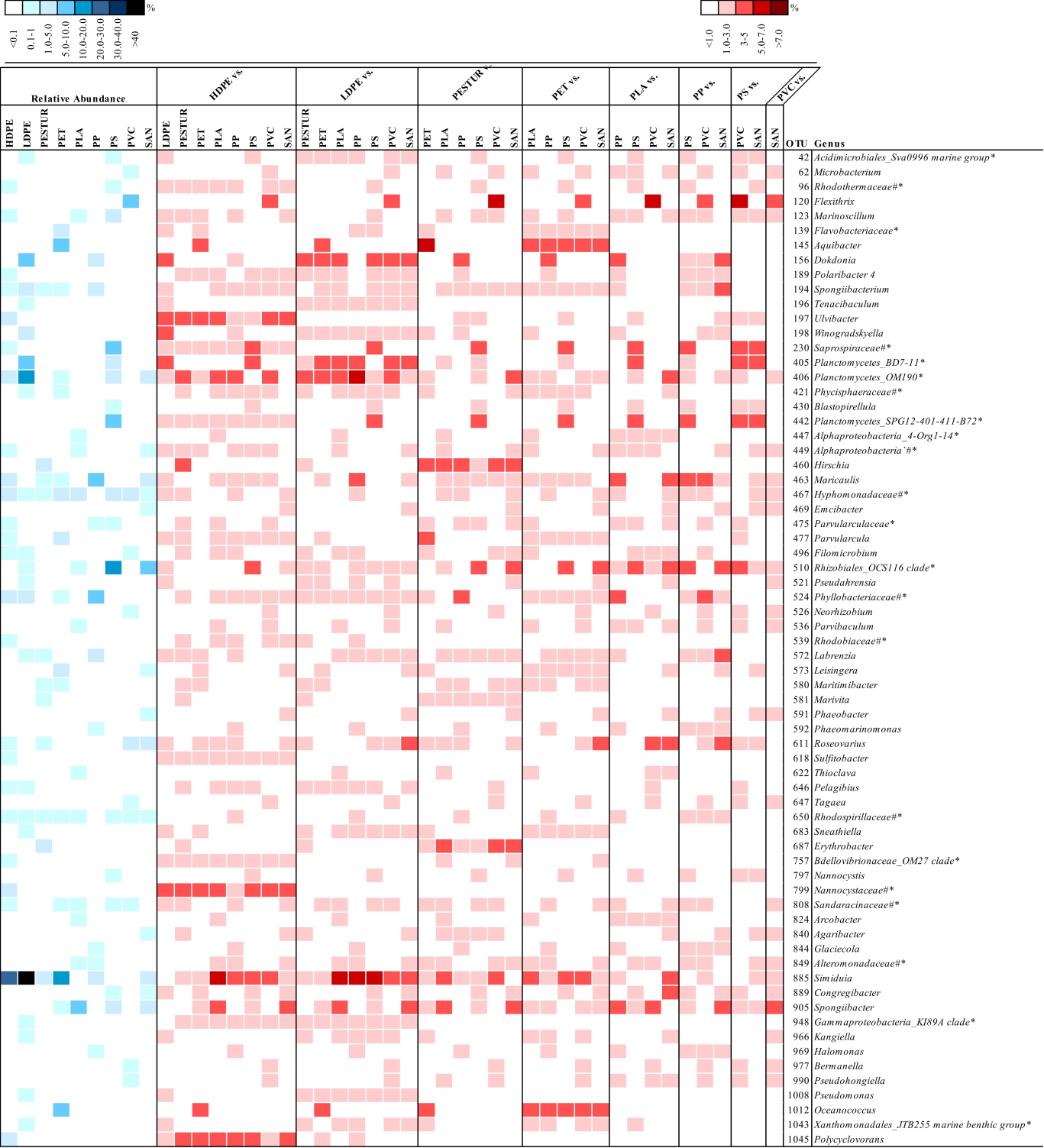

Supplement: S3 Fig — OTUs with a mean relative abundance of at least 0.1% (n = 5) in at least one substrate type were analysed. Displayed are OTUs jointly contributing to the total dissimilarity of at least 3% between plastic or with relative abundance of at least 1% on one substrate type. OTUs with a mean relative abundance of at least 0.1% present on both, plastics and glass, were rejected. The amount of contribution is indicated by the colour of cells, darker colours represent higher contributions. A * indicates the term “unclassified”, # indicates the term “uncultured”. (PDF) [file pone.0215859.s004.pdf]
